# Butyrate Attenuates Hepatic Steatosis Induced by a High‐Fat and Fiber‐Deficient Diet via the Hepatic GPR41/43‐CaMKII/HDAC1‐CREB Pathway

**DOI:** 10.1002/mnfr.202200597

**Published:** 2022-11-16

**Authors:** Mingxuan Zheng, Xiaoying Yang, Qingyuan Wu, Yuying Gong, Ning Pang, Xing Ge, Nathan Nagaratnam, Pengfei Jiang, Menglu Zhou, Tao Hu, Hui Hua, Kuiyang Zheng, Xu‐Feng Huang, Yinghua Yu

**Affiliations:** ^1^ Jiangsu Key Laboratory of Immunity and Metabolism Department of Pathogen Biology and Immunology Xuzhou Medical University Xuzhou Jiangsu 221004 P. R. China; ^2^ Tianjin Third Central Hospital Tianjin 300170 P. R. China; ^3^ Illawarra Health and Medical Research Institute (IHMRI) and School of Medicine University of Wollongong Wollongong NSW 2522 Australia; ^4^ National Experimental Demonstration Center for Basic Medicine Education Xuzhou Medical University Xuzhou Jiangsu 221004 P. R. China

**Keywords:** butyrate, CaMKII, CREB, GPR41, GPR43, HDAC1, NAFLD

## Abstract

**Scope:**

Hepatic steatosis is a major health issue that can be attenuated by a healthy diet. This study investigates the effects and molecular mechanisms of butyrate, a dietary fiber metabolite of gut microbiota, on lipid metabolism in hepatocytes.

**Methods and results:**

This study examines the effects of butyrate (0–8 mM) on lipid metabolism in primary hepatocytes. The results show that butyrate (2 mM) consistently inhibits lipogenic genes and activates lipid oxidation‐related gene expression in hepatocytes. Furthermore, butyrate modulates lipid metabolism genes, reduces fat droplet accumulation, and activates the calcium/calmodulin‐dependent protein kinase II (CaMKII)/histone deacetylase 1 (HDAC1)‐cyclic adenosine monophosphate response element binding protein (CREB) signaling pathway in the primary hepatocytes and liver of wild‐type (WT) mice, but not in G‐protein‐coupled receptor 41 (GPR41) knockout and 43 (GPR43) knockout mice. This suggests that butyrate regulated hepatic lipid metabolism requires GPR41 and GPR43. Finally, the study finds that dietary butyrate supplementation (5%) ameliorates hepatic steatosis and abnormal lipid metabolism in the liver of mice fed a high‐fat and fiber‐deficient diet for 15 weeks.

**Conclusion:**

This work reveals that butyrate improves hepatic lipid metabolism through the GPR41/43‐CaMKII/HDAC1‐CREB pathway, providing support for consideration of butyrate as a dietary supplement to prevent the progression of NAFLD induced by the Western‐style diet.

## Graphical abstract

Butyrate decreases body weight, improves insulin sensitivity and prevents hepatic steatosis induced by a high‐fat and fiber‐deficient diet. The underlying molecular mechanism of butyrate in preventing hepatic steatosis is attributed to activating the calcium/calmodulin‐dependent protein kinase II/histone deacetylase 1‐cyclic adenosine monophosphate response element binding protein (CaMKII/HDAC1‐CREB) signaling pathway via hepatic G‐protein‐coupled receptors 41 and 43.

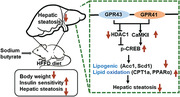

## 1 Introduction

Non‐alcoholic fatty liver disease (NAFLD) is the most common chronic liver disease worldwide, with a prevalence of about 25% in adults and up to 70% in patients with type 2 diabetes.^[^ ^1^, ^2^ ^]^ In addition, NAFLD is considered a leading cause of liver transplantation for end‐stage liver disease and hepatocellular carcinoma.^[^ ^3^ ^]^ Increased recognition of the importance of NAFLD in recent years has led to various treatment modalities, including lifestyle modification, pharmacological agents, and surgical intervention. Unfortunately, there are no effective therapies for NAFLD approved by Food and Drug Administration. Therefore, further research is required to identify an effective therapeutic approach or nutritional strategy for preventing and treating NAFLD.^[^ ^4^ ^]^

Butyrate is one of the short‐chain fatty acids (SCFAs) fermented from dietary fibers by gut microbiota.^[^ ^5^ ^]^ Previously, dietary supplementation of butyrate has been shown to prevent hepatic steatosis induced by a high‐fat diet in rodents^[^ ^6^, ^7^ ^]^ and db/db diabetic mouse model,^[^ ^8^ ^]^ in which the modulation of intestinal tight junctions and intestinal L cells to secret gut hormones, and regulation of gut‐liver axis have been investigated.^[^ ^7^, ^8^, ^9^ ^]^ Furthermore, it is reported that SCFAs, including butyrate released from the intestine, are taken up by the liver.^[^ ^10^, ^11^ ^]^ However, the direct effects of butyrate on hepatic lipid metabolism in hepatocytes and its underlying molecular mechanisms have not been elucidated.

G‐protein‐coupled receptors 41 and 43 (GPR41, GPR43) are homologous and belong to a family of orphan G protein‐coupled receptors that are tandemly encoded at a single chromosomal locus in humans and mice.^[^ ^12^ ^]^ GPR41 couples to Gi/o signaling pathways and is expressed in the peripheral nervous system, adipose tissue, intestine, and liver, while GPR43 couples to either Gi/o or Gq signaling pathways and is expressed in immune cells, adipose tissue, the intestine, and liver.^[^ ^12^, ^13^, ^14^ ^]^ Both GPR41 and GPR43 receptors can be activated by butyrate.^[^ ^15^ ^]^ For example, butyrate activates GPR41 and GPR43 to regulate lipolysis in adipocytes and cell physiology in yeast cells and HEK293T cells.^[^ ^12^ ^]^ Activation of GPR41 in cultured mouse adipocytes stimulates the expression of leptin, the hormone inhibiting appetite, and promoting negative energy metabolism.^[^ ^16^ ^]^ Butyrate activates GPR43 in the liver and improves hepatic glycogen metabolism in a mouse model of type 2 diabetes mellitus.^[^ ^17^ ^]^ GPR41 and GPR43 are highly expressed in the liver.^[^ ^18^ ^]^ However, the role of GPR41 and GPR43 in butyrate‐regulated lipid metabolism in hepatocytes is largely unknown. Furthermore, butyrate acts as a histone deacetylase (HDAC) inhibitor, participating in epigenetic gene regulation in cancer cells.^[^ ^19^ ^]^ Moreover, activation of GPR41 and GPR43 increases intracellular Ca^2+^,^[^ ^12^, ^20^ ^]^ which can further activate calcium/calmodulin‐dependent protein kinase II (CaMKII) ^[^ ^21^ ^]^ and phosphorylate cyclic adenosine monophosphate response element binding protein (CREB),^[^ ^22^, ^23^ ^]^ the transcription factor of genes for lipid metabolism in HAT22 cells.^[^ ^24^, ^25^ ^]^ This molecular signaling downstream of butyrate‐regulated lipid metabolism via GPR41 and GPR43 requires further investigation in hepatocytes.

Currently, the dietary pattern has been shifting from a traditional low‐fat, high‐fiber diet to a high‐fat, low‐fiber diet in Western countries, coinciding with a growing prevalence of obesity and metabolic disorders.^[^ ^26^, ^27^ ^]^ High consumption of fat intake has been associated with the risk of NAFLD.^[^ ^28^, ^29^ ^]^ A global diet survey showed that adults have a severely deficient dietary fiber intake, with 15 g day^−1^ being the average fiber intake per person in the United States,^[^ ^30^ ^]^ 13.6 g day^−1^ in the United Kingdom,^[^ ^31^ ^]^ and 11 g day^−1^ in China.^[^ ^32^ ^]^ This is significantly lower than the 25–35 g day^−1^ fiber intake recommended by the World Health Organization (WHO).^[^ ^33^ ^]^ Furthermore, a case‐control study shows that the daily dietary fiber intake was almost 50% lower in NAFLD patients.^[^ ^34^ ^]^ Therefore, in the present study, we used a combination of high‐fat and fiber deficient (HFFD) diets to mimic a Western‐style diet to induce the NAFLD mouse model. Using this novel model, we demonstrated that sodium butyrate ameliorated hepatic steatosis. Furthermore, using GPR41^−/−^ and GPR43^−/−^ mice, sodium butyrate's direct hepatic effect on lipid metabolism was shown by inhibiting lipid synthesis and activating lipid oxidation‐related gene expression via GPR41 and GPR43 receptors both in primary hepatocyte and the liver of HFFD mice. In particular, sodium butyrate activated the GPR41/43‐CaMKII/HDAC1‐CREB signaling pathway in the hepatocytes and liver of HFFD mice. Overall, we identified the hepatic lipid mechanistic insight into the anti‐NAFLD effects of butyrate.

## 2 Results

### 2.1 Sodium Butyrate Inhibited Lipogenic Genes and Activated Lipid Oxidation‐Related Gene Expression in Hepatocytes

**Figure 1:**
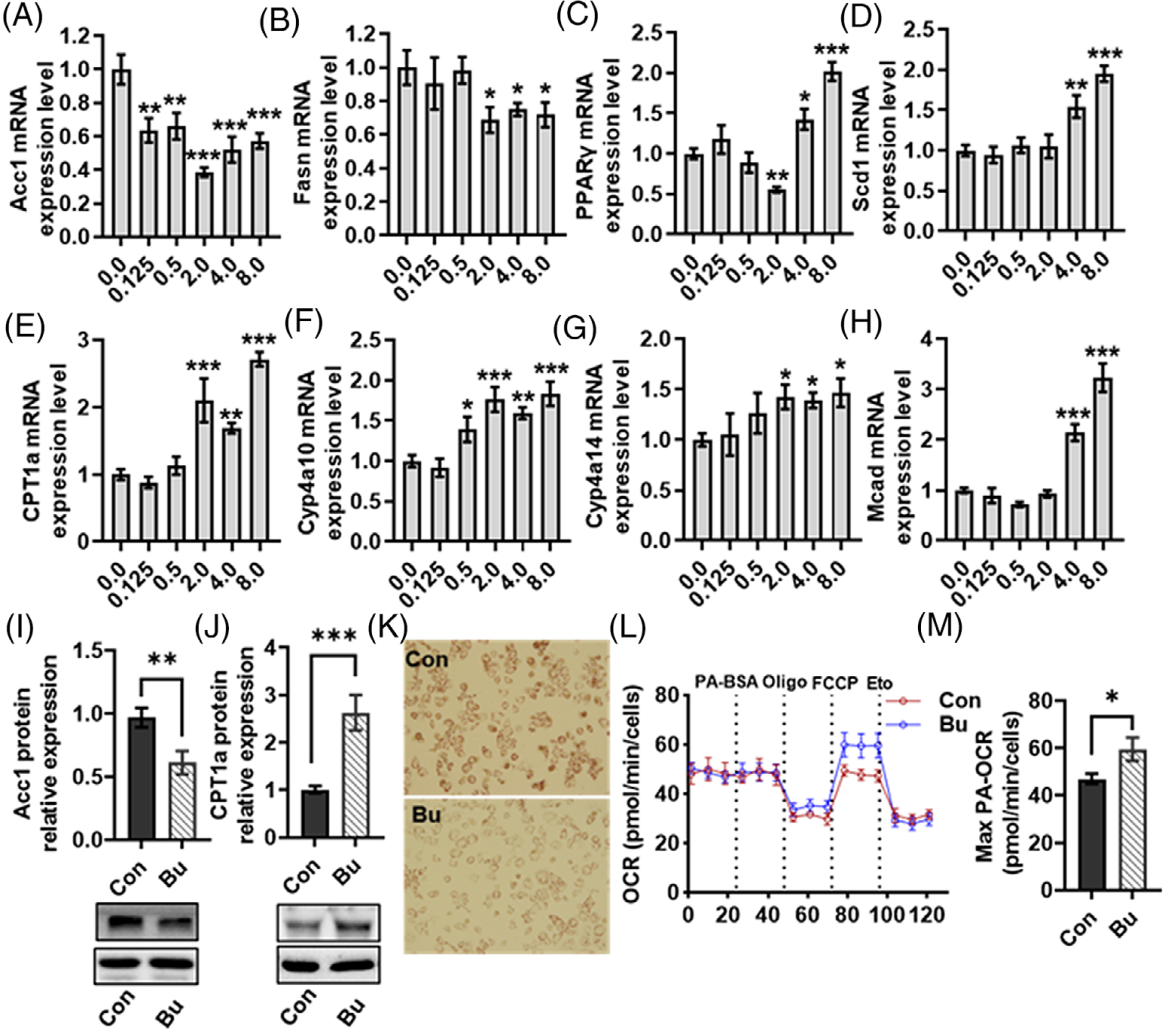
Sodium butyrate significantly inhibited lipogenic genes and activated lipid oxidation‐related gene expression in the primary hepatocytes. The mRNA expression of *Acc1* A), *Fasn* B), *PPARγ* C), and *scd1* D) related to lipogenesis and *CPT1a* E), *Cyp4a10* F), *Cyp4a14* G), and *Mcad* H) related to fatty acid oxidation were assessed by Real‐time PCR after treatment with sodium butyrate (0, 0.125, 0.25, 0.5, 1.0, 2.0, 4.0, and 8.0 mM) for 24 h. I–J) Protein expression of I) Acc1 and J) CPT1a in in primary hepatocytes cells of WT mice with or without 2 mM sodium butyrate. K) Representative images of Oil Red O staining in primary hepatocytes cells of WT mice with or without 2 mM sodium butyrate. L) Palmitate (PA) ‐OCR assay in the presence of sodium butyrate. Hep1‐6 were then treated sequentially with BSA‐conjugated palmitate (200 µM), oligomycin (Oligo) (2 µM), fluorocarbonyl cyanide phenylhydrazone (FCCP) (2.5 µM), and etomoxir (Eto) (50 µM) as indicated. M) The maximum PA‐OCR associated quantification. Values are means ± SEM (*n* = 6), *p < 0.05, **p < 0.01, ***p < 0.001 versus 0 mM. Con, Control group, Bu, 2 mM sodium butyrate.

Butyrate released from the intestine appears to be taken up by the liver.^[^ ^10^, ^11^ ^]^ However, the direct effects of butyrate on lipid metabolism in hepatocytes and its underlying molecular mechanisms have not been elucidated. In the present study, we explored the direct effects of sodium butyrate (0, 0.125, 0.25, 0.5, 1.0, 2.0, 4.0, and 8.0 mM) on lipogenesis and fatty acid oxidation in primary hepatocytes of WT mice (**Figure** **1**A–H). We found that sodium butyrate at 2 mM significantly inhibited the lipogenic genes acetyl‐CoA carboxylase 1 (*Acc1)* (*p* < 0.05, Figure 1A), fatty acid synthase (*Fasn)* (*p* < 0.05, Figure 1B), and peroxisome proliferator activated receptor γ (*PPARγ)* (*p* < 0.05, Figure 1C). In comparison, *PPARγ* and stearoyl coenzyme A desaturase (*Scd1)* expression were significantly increased by sodium butyrate at 4 and 8 mM *(p* < 0.05, Figure 1C,D). The mRNA expression of fatty acid oxidation‐related genes carnitine palmitoyl transferease 1a (*CPT1a)* (*p* < 0.05, Figure 1E), cytochrome P450, 4a10 (*Cyp4a10)* (*p* < 0.05, Figure 1F), and cytochrome P450, 4a14 (*Cyp4a14)* (*p* < 0.05, Figure 1G) were increased significantly by sodium butyrate at 2 mM. The medium‐chain acyl‐CoA dehydrogenase (*Mcad)* mRNA expression was increased by sodium butyrate at 4 and 8 mM (Figure 1H). Consistent with the above results, sodium butyrate 2 mM significantly increased CPT1a and decreased Acc1 at protein levels in the hepatocytes of WT mice (*p* < 0.05, Figure 1I,J). Moreover, oil red O staining showed that sodium butyrate inhibited lipid deposition in primary hepatocytes of WT mice (Figure 1K). Next, we examined if butyrate can inhibit fatty acid oxidation in hepatocytes in real‐time using the Seahorse XF24 Extracellular Flux Analyzer. Following this, palmitate (PA), a long‐chain fatty acid (LCFA), was supplemented into medium as a substrate. Butyrate significantly increased maximal PA‐associated oxygen consumption rate (OCR) (response to the uncoupling agent, FCCP) compared with the Con group in murine hepatocyte Hep1‐6 cells (Figure 1L,M). Furthermore, in response to the inhibition of LCFA transportation into mitochondria by CPT1 inhibitor etomoxir (Eto),^[^ ^35^ ^]^ this butyrate‐induced increase of PA‐associated OCR was abrogated (Figure 1L). Therefore, butyrate significantly induced up‐regulation of cellular OCR and fatty acid oxidation in the hepatocyte, in which CPT1 plays an important role. These observations support that butyrate directly affects hepatic lipid metabolism regulation. Furthermore, 2 mM sodium butyrate was chosen and used in the subsequent cell experiments to explore the molecular mechanism of sodium butyrate in regulating hepatic lipid metabolism.

### 2.2 Sodium Butyrate Regulated Hepatic Lipid Metabolism via GPR41 and GPR43 Receptors in the Primary Hepatocytes

**Figure 2:**
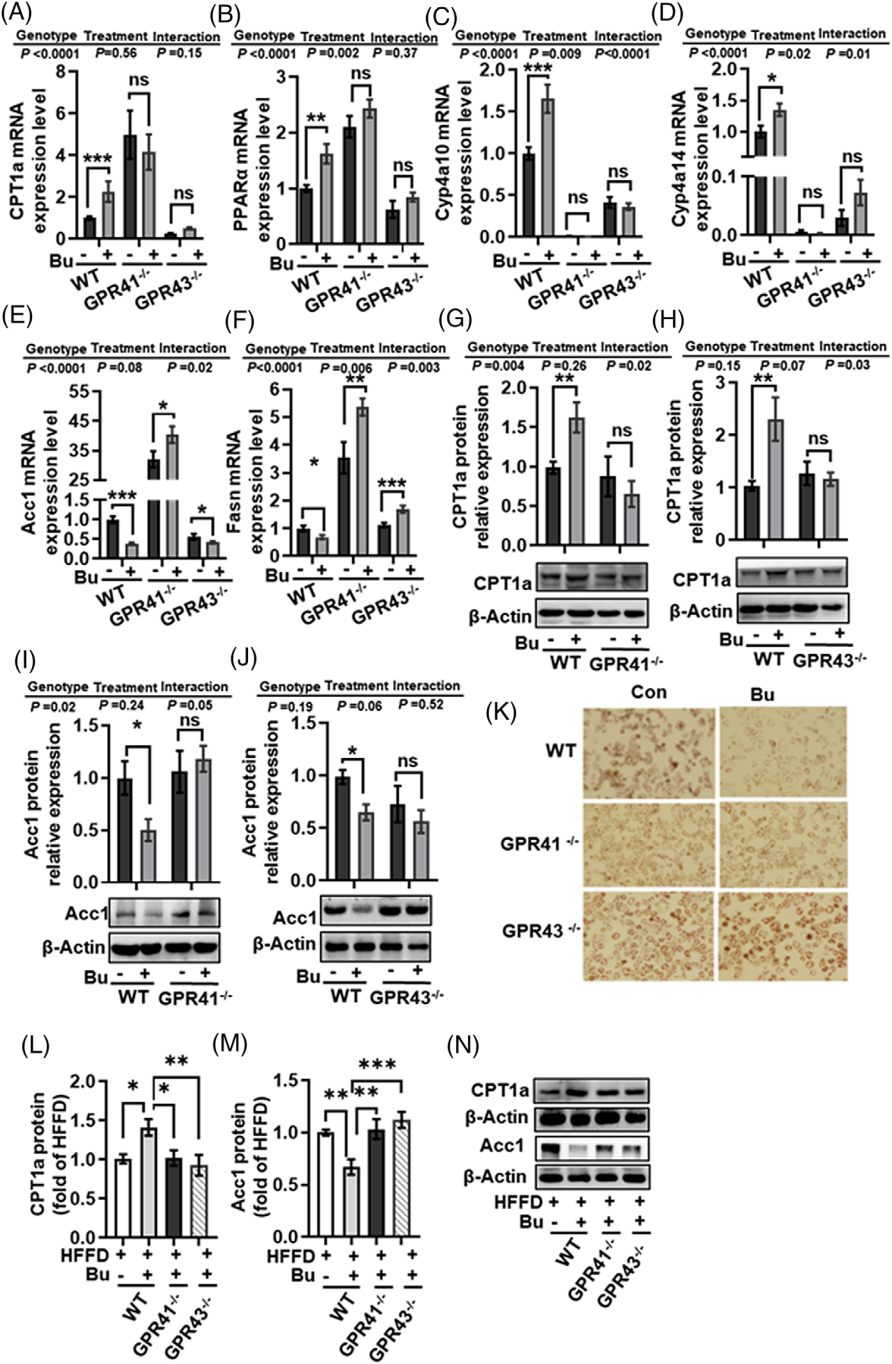
Sodium butyrate regulated hepatic lipid metabolism via the GPR41 and GPR43 receptors in the primary hepatocytes. A–D) The mRNA levels of *PPARα* A), *CPT1a* B), *Cyp4a10* C), and *Cyp4a14* D) related to fatty acid oxidation in the primary hepatocyte cells of wild‐type (WT), GPR41^−/−^ and GPR43*^−/−^* mice with or without 2 mM sodium butyrate. E, F) The mRNA levels of *Acc1* E) and *Fasn* F) are related to lipogenesis in the primary hepatocytes of wild‐type (WT), GPR41^−/−^ and GPR43*^−/−^* mice with or without 2 mM sodium butyrate. G–J) Protein expression of Acc1 and CPT1a in primary hepatocytes of wild‐type (WT), GPR41^−/−^, and GPR43*^−/−^* mice with or without 2 mM sodium butyrate. K) Representative images of Oil Red O staining in primary hepatocytes. L–M) Protein expression of CPT1a L) and Acc1 M) in the WT mice, GPR41^−/−^ and GPR43^−/−^ mice fed HFFD diet with or without sodium butyrate. N) Representative bands of lipid metabolism regulation proteins of liver tissue. Values are means ± SEM (*n* = 6), *p < 0.05, **p < 0.01, ***p < 0.001. Bu, sodium butyrate.

Butyrate can bind to GPR41 and GPR43 receptors^[^ ^12^ ^]^; however, the effects of these receptors in the hepatocyte have rarely been reported. To investigate the potential role of GPR41 and GPR43 in hepatocytes for butyrate‐regulated lipid metabolism, primary hepatocytes of *GPR41^−/−^* and *GPR43^−/‐^* mice were cultured with sodium butyrate for 24 h, followed by the detection of lipid deposition and lipid metabolism‐related genes and proteins expression. Firstly, knockdown of the GPR41 and GPR43 receptors was confirmed in the primary hepatocytes isolated from the liver tissue of *GPR41^−/−^* and *GPR43^−/^* ^−^ mice (*p* < 0.001, Figure S1A,B, Supporting Information). In the primary hepatocytes of *GPR41^−/−^* and *GPR43^−/‐^* mice, sodium butyrate did not increase the mRNA expression of genes related to fatty acid oxidation, including *PPARα* (*p* < 0.01, **Figure** **2**A), *CPT1a* (*p* < 0.001, Figure 2B), *Cyp4a10* (*p* < 0.001, Figure 2C), and *Cyp4a14* (*p* < 0.05, Figure 2D), which in contrast were up‐regulated by sodium butyrate in the primary hepatocytes of WT mice. For lipogenic genes, sodium butyrate significantly inhibited the mRNA expression levels of *Acc1* (*p* < 0.001, Figure 2E) and *Fasn* (*p* < 0.05, Figure 2F) in primary hepatocytes of WT mice; however, sodium butyrate did not inhibit *Acc1* and *Fasn* mRNA expression in primary hepatocytes of *GPR41^−/−^* mice, nor *Fasn* mRNA expression in primary hepatocytes *GPR43^−/‐^* mice. Furthermore, sodium butyrate increased CPT1a and decreased Acc1 protein levels in the hepatocytes of WT mice but not in *GPR41^−/−^* and *GPR43^−/‐^* mice (*p* < 0.05, Figure 2G–J). These results suggest that butyrate's regulation of hepatic lipid metabolism was mediated by hepatic GPR41 and GPR43. Consistently, Oil red O staining showed that sodium butyrate inhibited lipid deposition in primary hepatocytes of WT mice, while the inhibition effects were not present in *GPR41^−/−^* and *GPR43^−/‐^* mice (Figure 2K). These results indicate that GPR41 and GPR43 play a significant role in butyrate improving lipid metabolism in the primary hepatocyte.

We next investigated the response of the liver to sodium butyrate in vivo to gain insight into the mechanistic interactions between butyrate and GPR41/43. Sodium butyrate supplementation significantly increased CPT1a and inhibited Acc1 protein levels in the liver tissue of WT mice with HFFD diet for 21 weeks (*p* < 0.05, Figure 2L–N), but not in HFFD‐fed *GPR41^−/−^* and *GPR43^−/−^* mice.

### 2.3 Sodium Butyrate Activated the GPR41/43‐CaMKII‐CREB Signaling Pathway in Hepatocytes and Liver of HFFD Mice

**Figure 3:**
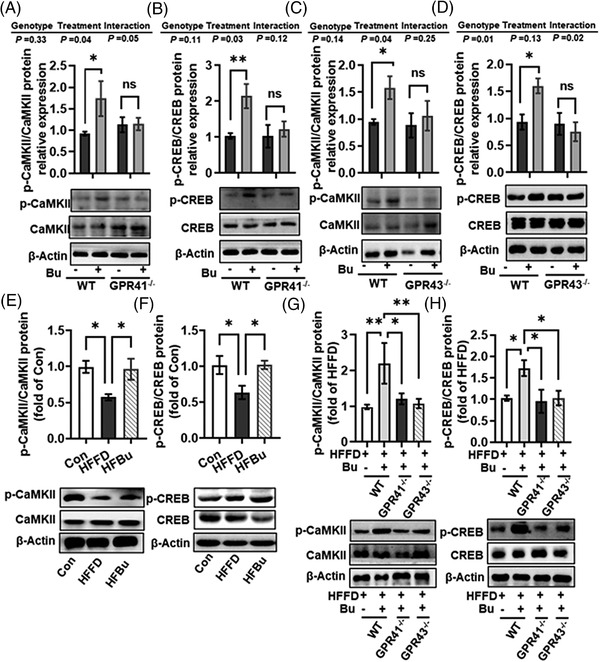
Sodium butyrate activated the GPR41/43‐CaMKII‐CREB signaling pathway in hepatocytes and liver. A–D) Protein expression of p‐CaMKII/CaMKII A, C) and p‐CREB/CREB B, D) in the primary hepatocytes of WT, GPR41^‐/‐^ and GPR43^‐/‐^ mice with or without 2 mM sodium butyrate. E, F) Protein expression of p‐CaMKII/CaMKII E) and p‐CREB/CREB F) in liver tissues of C57BL/6J mice fed a control (Con) diet, high‐fat fiber‐deficient diet (HFFD) diet, or HFFD diet with sodium butyrate (HFBu) diet for 21 weeks. G, H) Protein expression of p‐CaMKII/CaMKII G) and p‐CREB/CREB H) in the WT mice, GPR41^‐/‐^ and GPR43^‐/‐^ mice fed HFFD diet with or without sodium butyrate. Values are means ± SEM (*n* = 6), *p < 0.05, **p < 0.01, ***p < 0.001. Bu, sodium butyrate; ns, no significance.

Next, we studied the downstream cascades of GPR41 and GPR43 activated by butyrate in the hepatocytes. Both CaMKII and CREB were possible candidates. It has been reported that the activation of GPR41 and GPR43 results in augmented intracellular Ca^2+^,^[^ ^12^, ^20^ ^]^ where Ca^2+^ can activate CaMKII,^[^ ^21^ ^]^ which subsequently phosphorylates and activates CREB.^[^ ^22^, ^23^ ^]^ In primary hepatocytes, sodium butyrate significantly increased phosphorylation of CaMKII and, in turn, CREB in WT mice, but not in the *GPR41^−/−^* (**Figure** **3**A,B) and *GPR43^−/−^* mice (Figure 3C,D), suggesting that butyrate actives the CaMKII‐CREB signaling pathway via the GPR41 and GPR43 receptors.

Furthermore, in the liver, sodium butyrate supplementation prevented the decrease of phosphorylated CaMKII and CREB in the HFFD mice (*p* < 0.05, Figure 3E,F). However, sodium butyrate did not increase the phosphorylation of CaMKII and CREB in the liver of *GPR41^−/−^* and *GPR43^−/^* ^−^ mice following HFFD feeding (Figure 3G,H). These results suggest that butyrate activates the CaMKII‐CREB signaling via GPR41 and GPR43 receptors in the hepatocytes and the liver of HFFD mice.

### 2.4 Sodium Butyrate Regulated Lipid Metabolism through GPR41 and GPR43 Expression‐Dependent Inhibition of HDAC1

**Figure 4:**
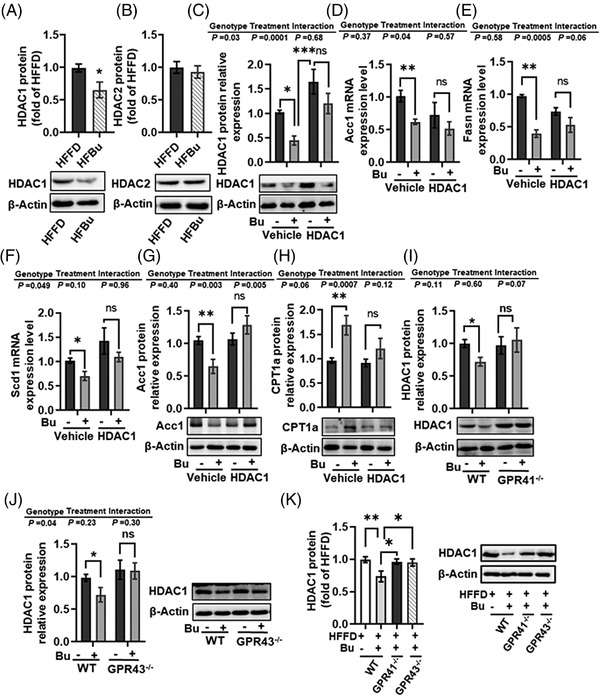
Sodium butyrate regulation of lipid metabolism through inhibition of HDAC1 is dependent on the expression of GPR41 and GPR43. A, B) Protein expression of HDAC1 A) and HDAC2 B) in liver tissue of the C57BL/6J mice fed a high‐fat fiber‐deficient diet (HFFD) with or without sodium butyrate for 21 weeks. C) HDAC1 in Hep1‐6 cells after over‐expression HDAC1 with or without sodium butyrate. The mRNA expression of Acc1 D), Fasn E), and Scd1 F), and protein expression of Acc1 G), and CPT1 H) in Hep1‐6 cells over‐expressed HDAC1 with or without sodium butyrate. I, J) Protein expression of HDAC1 in in primary hepatocytes cells of WT, GPR41^‐/‐^ and GPR43^‐/‐^ mice with or without 2 mM sodium butyrate. K) Protein expression of HDAC1 in the WT mice, GPR41^‐/‐^ and GPR43^‐/‐^ mice fed HFFD diet with or without sodium butyrate. Values are means ± SEM (*n* = 6), *p < 0.05, **p < 0.01, ***p < 0.001. Bu, sodium butyrate; ns, no significance.

Among the SCFAs, butyrate is the most potent in inhibiting the activities of HDAC1 and HDAC2.^[^ ^36^, ^37^, ^38^ ^]^ Therefore, we examined the effects of sodium butyrate on HDAC1 and HDAC2 in the liver during HFFD diet feeding. We found that HDAC1, but not HDAC2 levels were significantly decreased in the liver of the HFBu group compared with the HFFD group (*p* < 0.05, **Figure** **4**A,B), suggesting HDAC1 plays an essential role in butyrate‐regulated hepatic lipid metabolism. It is reported that HDAC1 blocks the phosphorylation of CREB,^[^ ^39^ ^]^ a transcription factor regulating lipogenic genes.^[^ ^24^, ^25^ ^]^ Therefore, we examined the potential role of HDAC1 in sodium butyrate's regulation of lipogenic genes using the HDAC1 overexpression plasmid to transfect Hep1‐6 cells. Firstly, sodium butyrate decreased the HDAC1 level in control Hep1‐6 cells but not in HDAC1‐overexpression cells (Figure 4C). Furthermore, sodium butyrate did not significantly inhibit the mRNA expression of lipogenic genes, including the *Acc1* (*p* < 0.01, Figure 4D), *Fasn* (*p* < 0.001, Figure 4E), and *Scd1* (*p* < 0.05, Figure 4F) in HDAC1‐overexpression cells, but did so in the control cells. Regarding protein levels, sodium butyrate inhibited Acc1 (*p* < 0.05, Figure 4G) and promoted CPT1a (*p* < 0.05, Figure 4H) in control cells but not in HDAC1‐overexpression cells, suggesting inhibition of HDAC1 is needed for sodium butyrate's regulation of lipid metabolism in hepatocytes. Although butyrate activates GPR41 and GPR43 and inhibits HDAC1,^[^ ^12^, ^19^ ^]^ it is unknown whether butyrate regulates HDAC1 via GPR41 and GPR43 in hepatocytes. We found that sodium butyrate significantly decreased HDAC1 levels in the primary hepatocytes of WT mice but not in the *GPR41^−/−^* and *GPR43^−/−^* mice (Figures 4I,J). In addition, sodium butyrate supplementation inhibited the HDAC1 level in the liver tissue of HFFD‐fed WT mice but not in HFFD‐fed *GPR41^−/‐^* and *GPR43^−/−^* mice (Figure 4K). Collectively, these results show that HDAC1 and the downstream signaling molecule CREB are important for sodium butyrate GPR41‐ and GPR43‐dependent improvement of lipid metabolism in hepatocytes.

### 2.5 Sodium Butyrate Ameliorated Hepatic Steatosis and Abnormal Lipid Metabolism in the Liver of HFFD Mice

**Figure 5:**
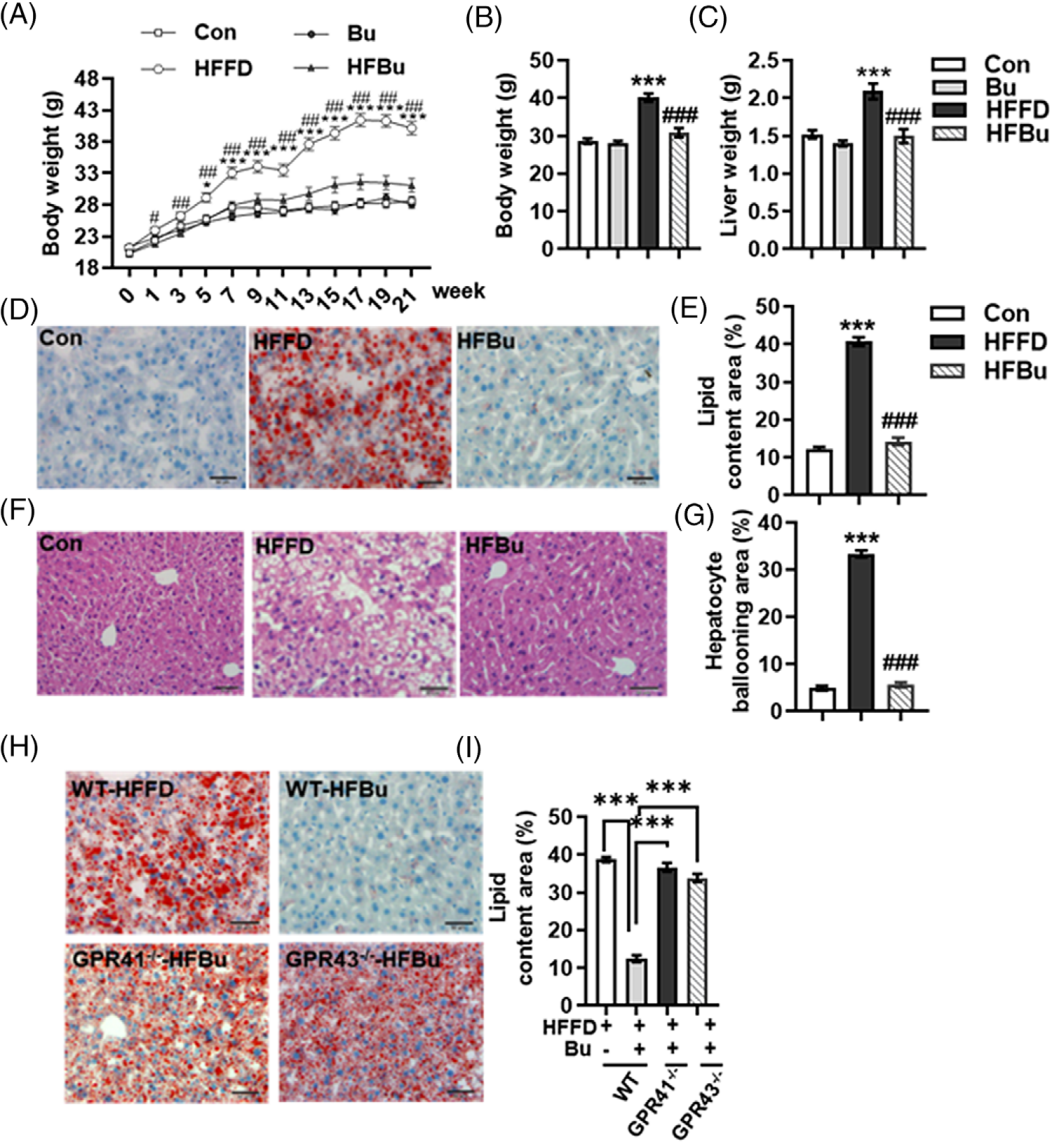
Sodium butyrate ameliorated hepatic steatosis induced by a high‐fat and fiber‐deficient (HFFD) diet. A) The body weight during 21 weeks of intervention and B) the final body weight of C57BL/6J mice fed a control (Con) diet, control diet supplement with sodium butyrate (Bu), HFFD diet, or HFFD diet with sodium butyrate (HFBu) diet for 21 weeks (*n* = 10). C) The liver weight. D, E) Representative histological images and quantification of hepatic lipid area of frozen liver sections after Oil Red O staining in Con, HFFD, and HFBu of WT mice (*n* = 3). F, G) Representative histological images and quantification of hepatic ballooning of frozen liver sections after H&E staining in Con, HFFD, and HFBu of WT mice (*n* = 3). H, I) Representative histological images and quantification of hepatic lipid area of frozen liver sections after Oil Red O staining in WT mice fed with HFFD or HFBu, GPR41^−/−^ fed with HFBu, and GPR43^−/−^ fed with HFBu (*n* = 3). *p < 0.05, **p < 0.01, ***p < 0.001 versus Con mice; ^#^ *p* < 0.05, ^##^ *p* < 0.01, ^###^ *p* < 0.001 versus HFFD mice.

**Figure 6:**
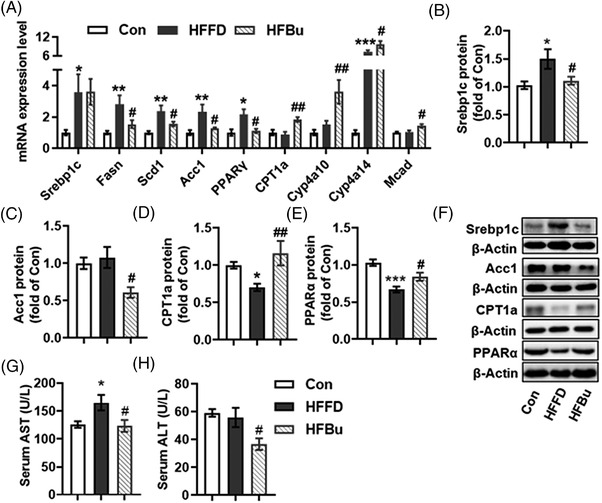
Sodium butyrate inhibited lipogenesis and increased fatty acid oxidation. A) The mRNA expression of lipogenic genes (Srebp1c, Fasn, Scd1, Acc1, and PPARγ) and fatty acid oxidation genes (PPARα, CPT1a, Cyp4a10, Cyp4a14, and Mcad) in liver tissue. Protein expression of Srebp1c B) and Acc1 C) related to lipogenesis in liver tissue. Protein expression of CPT1a D) and PPARα E) related to fatty acid oxidation in liver tissue (*n* = 6). F) Representative bands of lipid metabolism regulation proteins of liver tissue. G, H) Serum AST and ALT. Values are means ± SEM, *p < 0.05, **p < 0.01, ***p < 0.001 versus Con mice; ^#^ *p* < 0.05, ^##^ *p* < 0.01, ^###^ *p* < 0.001 versus HFFD mice.

Finally, we investigated whether sodium butyrate prevented hepatic steatosis induced by a diet of high fat and fiber‐deficiency. The HFFD group had a significantly higher body and liver weight compared to the control group. In contrast, sodium butyrate supplementation significantly prevented this body weight gain (*p* < 0.05, **Figure** **5**A,B) and reduced liver weight (*p* < 0.05, Figure 5C). Sodium butyrate alone did not alter body weight and liver weight. The Oil Red and H&E‐ staining showed that mice fed the HFFD diet had significantly greater hepatic lipid deposits inside the parenchyma cells and hepatocyte ballooning compared with the control group (Figure 5D–G). While sodium butyrate supplementation prevented this hepatic steatosis and injury, sodium butyrate supplementation did not ameliorate hepatic steatosis in the liver of *GPR41^−/−^* and *GPR43^−/^* ^−^ mice with HFFD diet (*p* < .001, Figure 5H,I). To further investigate the genetic regulatory effects of sodium butyrate on lipid metabolism, the expression of fatty acid synthesis and oxidative genes in the liver were detected using RT‐PCR. The mRNA expression of genes related to lipogenesis (s*terol regulatory element‐binding transcription factor 1c (Srebp1c), Fasn, Scd1, Acc1*, and *PPARγ*) were up‐regulated in the liver of the HFFD group (*p* < 0.05, **Figure** **6**A). Sodium butyrate prevented this up‐regulation of *Fasn*, *Scd1*, *Acc1*, and *PPARγ* mRNA expression. Furthermore, the mRNA expression of fatty acid oxidation related genes (*CPT1a*, *Cyp4a10*, *Cyp4a14*, and *Mcad*) was significantly up‐regulated in the HFBu group compared with the HFFD group (*p* < 0.05, Figure 6A). Protein levels of Srebp1c and Acc1 were also significantly reduced in the HFBu group compared to the HFFD group (*p* < 0.05, Figure 6B,C,G), and the fatty acid oxidation related proteins (CPT1a and PPARα) were significantly up‐regulated in the HFBu group (*p* < 0.05, Figure 6D–G). Compared with the HFFD group, the serum aspartate transaminase (AST) and alanine aminotransferase (ALT) levels were reduced by sodium butyrate (*p* < 0.05, Figure 6H,I), indicating that butyrate had a protective effect from liver damage.

**Figure 7:**
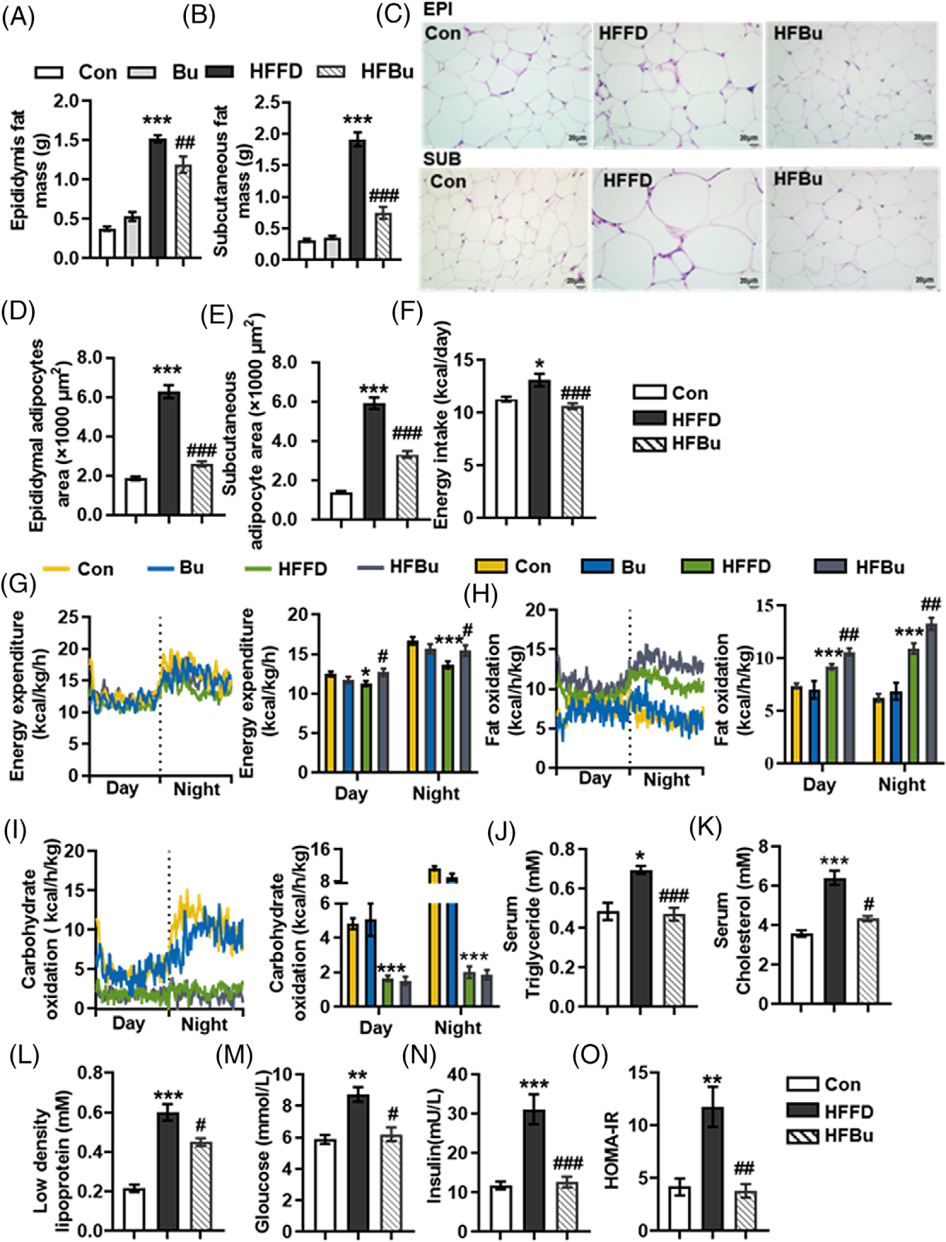
Sodium butyrate attenuated fat accumulation and improved metabolic parameters in mice with a high‐fat fiber deficient (HFFD) diet. A–C) The epididymis fat A) and subcutaneous fat B) of C57BL/6J mice fed a control (Con) diet, control diet supplement with sodium butyrate (Bu), HFFD diet or HFFD diet with sodium butyrate (HFBu) diet for 21 weeks (*n* = 10). C–E) The epididymal adipocytes area C, D) and subcutaneous adipocytes area C, E) were quantified after H&E staining (*n* = 3). F) Energy intake. The mice were housed in fully automated metabolic cages in which G) energy expenditure was measured. H) Fat oxidation and I) carbohydrate oxidation were calculated. J–L) Serum triglycerides, cholesterol, and low‐density lipoprotein. M–O) Serum glucose, insulin, and HOMA‐IR (*n* = 10). Values are means ± SEM, *p < 0.05, **p < 0.01, ***p < 0.001 versus Con mice; ^#^ *p* < 0.05, ^##^ *p* < 0.01, ^###^ *p* < 0.001 versus HFFD mice. EPI, epididymal fat; SUB, subcutaneous fat.

In addition, sodium butyrate intervention attenuated fat accumulation in mice on the HFFD diet (*p* < 0.05, **Figure** **7**A–E). Moreover, sodium butyrate supplementation inhibited the increased energy intake observed in HFFD mice (*p* < 0.05, Figure 7F). The indirect calorimetry analysis showed that sodium butyrate significantly increased the whole‐body oxygen consumption (VO2) and carbon dioxide production (VCO2), which were significantly lower in the HFFD group than in the control group for both day and night (*p* < 0.05, Figure S2A,B, Supporting Information). Accordingly, sodium butyrate increased energy expenditure (*p* < 0.05, Figure 7G) and fat oxidation (*p* < 0.05, Figure 7H). However, there was no statistically significant difference in carbohydrate oxidation between the HFBu group and the HFFD group (Figure 7I). The serum triglyceride, cholesterol, and low‐density lipoprotein levels were significantly higher in the HFFD group compared with the control group (*p* < 0.05, Figure 7J–L). Sodium butyrate restored these lipid metabolic parameters (*p* < 0.05, Figure 7J–L). We also found that sodium butyrate improved glucose metabolism by decreasing fasting serum glucose, insulin, and homeostasis assessment model insulin resistance ( HOMA‐IR) (*p* < 0.05, Figure 7M–O).

Collectively, these results indicate that sodium butyrate supplementation attenuated hepatic steatosis, improved lipid and glucose metabolism, and increased energy expenditure in mice fed an HFFD diet; however, GPR41 and GPR43 receptor knockout blocks the sodium butyrate attenuation of hepatic steatosis.

## 3 Discussion

**Figure 8:**
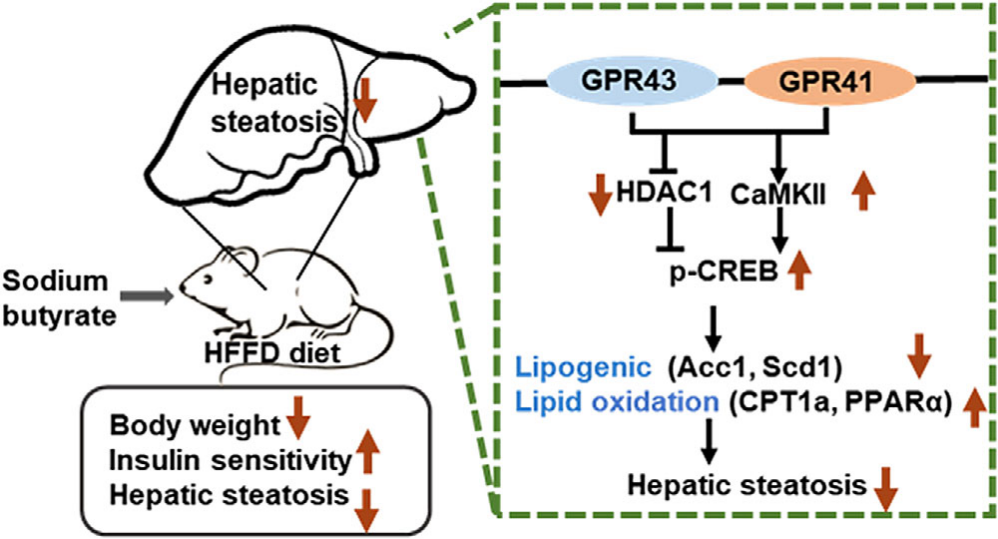
Schematic representation of the mechanism by which sodium butyrate improves hepatic steatosis and abnormal lipid metabolism in the liver of mice fed a high‐fat and fiber‐deficient (HFFD) diet. Sodium butyrate consistently inhibited lipogenic genes and activated lipid oxidation‐related genes in hepatocytes. The underlying molecular mechanism has been determined as sodium butyrate activates the CaMKII‐CREB and inhibits the HDAC1‐CREB signaling pathway via hepatic GPR41/43.

In the present study, we demonstrated that sodium butyrate ameliorated hepatic steatosis induced by a diet of high fat and fiber‐deficiency. Sodium butyrate's direct effect on hepatic lipid metabolism was shown by inhibiting lipogenic genes and activating lipid oxidation‐related gene expression in hepatocytes via GPR41 and GPR43 receptors. In addition, sodium butyrate activated the GPR41/43‐CaMKII/HDAC1‐CREB signaling pathway both in primary hepatocytes and the liver of HFFD mice. Overall, we showed sodium butyrate's protective effects on hepatic steatosis and identified the novel underlying mechanisms in hepatocytes for preventing NAFLD progression induced by a Western‐style diet consisting of high‐fat and fiber deficiency (**Figure** **8**).

Previously, most studies have induced a mouse model of NAFLD through a chronic high‐fat diet.^[^ ^40^, ^41^ ^]^ Currently, both high fat and fiber deficiency are considered two essential characteristics of the Western‐style diet, strongly associated with obesity and related metabolic diseases.^[^ ^42^ ^]^ Accordingly, to mimic the current most common Western dietary pattern, we used the HFFD diet to induce hepatic steatosis and injury, hypertriglyceridemia, and hypercholesterolemia in mice with alterations of hepatic genes related to fatty acid synthesis and oxidation. Importantly, we found that dietary supplementation with sodium butyrate ameliorated hepatic steatosis induced by the HFFD diet. Previously, dietary butyrate supplementation has been shown to prevent or reverse hepatic steatosis, both induced by high‐fat diet‐fed rodents^[^ ^6^, ^7^ ^]^ and in db/db mice,^[^ ^8^ ^]^ in which the potential mechanisms considered are butyrate modulating intestinal tight junctions to improve gut‐liver axis^[^ ^8^, ^9^ ^]^ or increased satiety hormone in the intestine reducing appetite via the gut‐brain neural circuit.^[^ ^7^ ^]^ Notably, the gut is the main organ for butyrate production, while the liver can subsequently take up virtually all butyrate.^[^ ^10^ ^]^ After being released into the portal vein, butyrate reaches the liver, the vital organ for lipid metabolism.^[^ ^43^ ^]^ In this study, we found that in primary hepatocytes, sodium butyrate inhibited lipid deposition, inhibited lipid synthesis‐related gene expression (*Acc1*, *Fasn*, *Scd1*, and *PPARγ*), and activated lipid oxidation‐related gene expression (*CPT1a*, *Cyp4a10*, and *Cyp4a14*). These findings suggest that the direct effects of butyrate‐regulated lipid metabolism in hepatocytes (inhibiting lipogenesis and increasing fatty acid oxidation) may be responsible for the anti‐NAFLD effects.

Butyrate is considered a ligand of GPR41 and GPR43,^[^ ^12^ ^]^ which are highly expressed in the liver tissue.^[^ ^44^ ^]^ However, it has not previously been reported whether sodium butyrate regulates lipid metabolism via GPR41 and GPR43 receptors of the liver. In the present study, using primary hepatocytes from *GPR41^−/−^* and *GPR43^−/‐^* mice, we found that GPR41 and GPR43 are vital for butyrate's regulation of hepatic lipid metabolism. Sodium butyrate inhibited lipogenic gene expression and activated fatty acid oxidation‐related genes in the primary hepatocytes of WT mice but not in *GPR41^−/−^* and *GPR43^−/‐^* mice. Furthermore, in the liver tissue, we also found that sodium butyrate supplementation significantly enhanced lipid oxidation and inhibited fatty acid synthesis in WT mice fed the HFFD diet for 21 weeks but not in *GPR41^−/−^* and *GPR43^−/−^* mice. This indicates that GPR41 and GPR43 play essential roles in butyrate's prevention of the abnormal hepatic lipid metabolism induced by the Western‐style diet.

CaMKII, a major mediator of Ca^2+^ signaling in cells,^[^ ^45^ ^]^ is reported to regulate lipid metabolism.^[^ ^46^ ^]^ The activation of CaMKII elevates fatty acid oxidation‐related gene *CPT1* expression and reduces the lipid synthesis gene *Acc1* expression in rat skeletal muscle.^[^ ^47^ ^]^ In the present study, we found that the phosphorylation levels of CaMKII in the liver were decreased in the HFFD mice; however, sodium butyrate supplementation prevented the attenuation of p‐CaMKII, suggesting that butyrate‐activated CaMKII contributes to cascades of metabolism in liver tissue, shifting from lipogenesis to fatty acid oxidation. Furthermore, in the primary hepatocytes, butyrate increased p‐CaMKII in WT mice but not in the *GPR41^−/−^* and *GPR43^−/−^* mice. This indicates that butyrate activates CaMKII by GPR41 and GPR43 receptors in the hepatocytes. Previously, it has been reported that the GPR41 and GPR43 heteromer enhances intracellular Ca^2+^ influx,^[^ ^48^ ^]^ which binds to CaMKII.^[^ ^21^ ^]^ Moreover, CaMKII activates and phosphorylates CREB on Ser133.^[^ ^22^, ^23^, ^24^ ^]^ CREB is an important transcriptional regulator of hepatic lipogenic genes, including PPARγ and Srebp1c.^[^ ^24^, ^25^ ^]^ For instance, mice deficient in CREB activity have an increased PPARγ expression and show a fatty liver phenotype.^[^ ^25^ ^]^ In the present study, we found that sodium butyrate intervention increased the phosphorylation of CREB and prevented the alterations of PPARγ and Srebp1c in the liver tissue of mice. Collectively, these findings indicate that activation of the GPR41/43‐CaMKII‐CREB signaling pathway in hepatocytes is an important molecular mechanism in butyrate ameliorating NAFLD induced by a Western‐style diet.

It has been reported that HDAC1 and HDAC2 are involved in lipid metabolism.^[^ ^49^, ^50^, ^51^ ^]^ For example, the genetic deletion of both *Hdac1* and *Hdac2* in mouse embryonic fibroblasts results in decreased lipid accumulation.^[^ ^52^ ^]^ Treatment with HDAC1/2 inhibitors leads to activation of fatty acid oxidation, with increases in genes related to catabolism of fatty acids, β‐oxidation, and fatty acid transporters.^[^ ^53^ ^]^ Butyrate is an HDACs inhibitor, significantly inhibiting HDAC1 and HDAC2.^[^ ^54^ ^]^ We found that sodium butyrate significantly suppressed HDAC1 but not HDAC2 levels in the liver tissue of mice fed the HFFD diet. Furthermore, sodium butyrate decreased HDAC1 levels in the Hep1‐6 cells but failed to reduce HDAC1 levels significantly in the HDAC1‐overexpression cells. Therefore, butyrate's inhibition of HDAC1 may contribute to lipid metabolism in the liver. Previously, Wang et al. found that HDAC1‐overexpressed mice show increased lipid deposition in the liver, but the underlying mechanism has not been elucidated.^[^ ^55^ ^]^ Research shows that HDAC1 blocks Ser133 phosphorylation of CREB,^[^ ^39^ ^]^ the same site activated by CaMKII,^[^ ^22^, ^23^, ^24^ ^]^ suggesting HDAC1 and CaMKII both regulate CREB with opposite effects on lipid metabolism regulation. Indeed, we found that overexpression of HDAC1 abolished sodium butyrate inhibition of lipogenesis gene mRNA expression and protein level in Hep1‐6 cells, indicating activation of HDAC1 may cause a shift toward lipid anabolism. However, previous research shows that activation of CaMKII elevates fatty acid oxidation and inhibits lipid synthesis.^[^ ^47^ ^]^ It has been reported that butyrate modulates biological responses by binding to the GPR41 and GPR43 receptors, as well as inhibiting HDAC1^[^ ^12^, ^19^ ^]^; however, until now, it has been unclear in hepatocytes if HDAC1 is regulated by GPR41 and GPR43. In the present study, sodium butyrate decreased HDAC1 expression in primary hepatocytes of WT mice but not in *GPR41^−/−^* and *GPR43^−/‐^* mice, suggesting the inhibitory effect of butyrate on HDAC1 is dependent on GPR41 and GPR43. Furthermore, since sodium butyrate's activation of CREB was blocked following GPR41 and GRP43 knockout, butyrate's activation of CREB is also dependent on GPR41 and GRP43. Collectively, this suggests that butyrate may improve lipid metabolism through inhibition of HDAC1‐CREB via activation of GPR41 and GRP43 in hepatocytes.

In this study, the effects of butyrate on steatohepatitis improvement were only examined in male obese mice. The study was conducted in male mice because it has been reported that male mice are more likely to develop hepatic steatosis than female mice owing to estrogen's protective effects against NAFLD.^[^ ^56^, ^57^ ^]^ For example, the female liver clears fatty acids from plasma more rapidly than the male liver due to an enhanced transport rate through the cell membrane of hepatocytes.^[^ ^58^, ^59^ ^]^ Furthermore, the overall NAFLD prevalence is lower in women than in men. For example, premenopausal women were found to have lower NAFLD prevalence and incidence than age‐matched men.^[^ ^60^, ^61^ ^]^ However, the prevalence of NAFLD becomes similar in men and women after menopause,^[^ ^62^, ^63^, ^64^ ^]^ which is considered to be due to the protective effect of estrogen.^[^ ^65^ ^]^ Therefore, in the present study, we examined the effects of butyrate in male mice to avoid the effects of estrogen and ovaries on hepatic fatty acid synthesis. In a future study, the effects of butyrate on steatohepatitis in females should be further investigated.

In summary, the current study demonstrated that sodium butyrate has a hepatoprotective effect on NAFLD induced by a Western‐style diet consisting of high fat and fiber deficiency. The underlying molecular mechanism of sodium butyrate in preventing hepatic steatosis may be attributed to the activation of CaMKII‐CREB and inhibition of the HDAC1‐CREB signaling pathway via hepatic GPR41/43. Overall, these findings suggest that butyrate supplementation or butyrate‐producing prebiotics or probiotics could be a therapeutic strategy to counteract Western‐style diet‐related NAFLD.

## 4 Experimental Section

### Cell Culture

Primary hepatocytes were isolated from the livers of WT, GPR41^−/−^, and GPR43^−/−^ mice (8–12 weeks old in C57BL/6J background) and cultured as previously reported.^[^ ^66^ ^]^ Briefly, mice were anesthetized, and their livers were perfused with 0.5 mg mL^−1^ type II collagenase (Gbico) via the inferior vena cava and isolated into hepatocytes. Primary hepatocytes were cultured in RPMI‐1640 (Hyclone) supplemented with 10% fetal bovine serum (FBS) and 1% penicillin‐streptomycin at 37 °C in the incubator and harvested 24 h after sodium butyrate administration. Sodium butyrate (B5887) for cell experiments was purchased from Sigma‐Aldrich. Murine hepatocyte Hep1‐6 cells were purchased from the Cell Bank of Shanghai Institute of Biological Science (SIBS, CAS, Shanghai, China). The cells were cultured in a high‐glucose medium (Hyclone) supplemented with 10% FBS and 1% penicillin‐streptomycin at 37 °C in the incubator.

### Fatty Acid Oxidation (FAO) Assay

For the FAO assay, the Seahorse XF24 Extracellular Flux Analyzer (Agilent), Palmitate‐BSA reagent (GLPBIO), Mito Stress Test Kits (Agilent), and etomoxir (Eto) (GLPBIO) were used to assess the cell's ability to oxidize exogenous fatty acid as per manufacturers’ instructions with minor modifications. All results were analyzed using Wave software version 2.4.0 (Agilent).

In brief, Hep1‐6 cells (2.0 × 10^4^) were seeded into 24‐well Seahorse microplates in 250 µL of growth medium and incubated at 37 °C, 5% CO_2_ overnight. To measure FAO, Hep1‐6 cells were cultured in substrate‐limited DMEM containing 0.5 mM glucose, 1 mM GlutaMAX, 0.5 mM carnitine, and 1% FBS and treated with or without butyrate for 16 h. Before starting the test, the cells were washed twice and equilibrated with KHB buffer (pH 7.4, 110 mM NaCl, 4.7 mM KCl, 2 mM MgSO_4_, 1.2 mM Na_2_HPO_4_, 2.5 mM glucose, and 0.5 mM carnitine) and incubated at 37 °C for 1 h without CO_2_. To induce FAO, palmitate‐BSA complex was injected at a final concentration of 200 µM into the XF‐24 cartridge. FAO capacity was represented as increased oxygen consumption rate (OCR) in response to the palmitate‐BSA complex. OCR was measured under basal state and in response to 2 µM oligomycin (Oligo) (Agilent), 2.5 µM fluorocarbonyl cyanide phenylhydrazone (FCCP) (Agilent), and 50 µM Eto (GLPBIO) employing the Seahorse XF24 Extracellular Flux Analyzer (Agilent) using Wave software, version 2.4.0 (Agilent).

### Cell Transfection

The Hep1‐6 cells (5 × 10^5^ per well) were seeded into six‑well plates. On reaching 80% confluence, the cells were maintained in serum‑free Opti‑MEM I medium (Thermo Fisher Scientific, Inc.). They were then transfected with 2.5 µg of expression vector pCMV‑HDAC1 (Cat: MG53562‐CF, Sino Biological, Beijing, China) containing mouse HDAC1 cDNA and 5 µL Lipofectamine 3000 reagent (Thermo Fisher Scientific, Inc.) premixed with serum‑free Opti‑MEM I media. The transfected cells were incubated in a CO_2_ incubator at 37 °C for 4 h, after which the medium was replaced with standard DMEM containing 10% FBS. Following transfection for 24 h, the stably transfected cells were administered with sodium butyrate for another 24 h.

### Animals and Treatment

WT male C57BL/6J mice aged 7 weeks were purchased from the Experimental Animal Center of Xuzhou Medical University (Xuzhou, China, SCXK (Su) 2015‐0009). GPR41^−/−^ and GPR43^−/−^ mice were purchased from BIORAY LABORATORIES Inc. (Shanghai, China). All animals were housed and maintained in a 12‐h light/dark photoperiod with unrestricted water and food. All animal care and experiments were carried out following protocols approved by the ethics committee of Xuzhou Medical University. After habituation to the laboratory environment for 1 week, the WT male mice were randomly divided into four groups (*n* = 10 per group): 1) the control group (Con) were fed a lab chow (LabDiet 5010, 5% fat by weight, 15% neutral detergent fiber by weight); 2) the butyrate (Bu) group were fed a diet of sodium butyrate mixed into lab chow diet (5% butyrate by weight); 3) the HFFD group were fed a diet with high fat (31.5% fat by weight) and fiber deficient (5% fiber by weight) same as the previous study^[^ ^67^ ^]^; 4) the HFBu group were fed an HFFD diet supplemented with sodium butyrate, at 50 g kg^−1^ sodium butyrate (5% butyrate by weight) (diet details were outlined in Table S1, Supporting Information). According to a previous study, a dosage of 5% sodium butyrate reduced appetite and activated brown adipose tissue via the gut‐brain neural circuit.^[^ ^7^ ^]^ Sodium butyrate (S817488) for the animal experiment was purchased from Shanghai Macklin Biochemical Co., Ltd. (China). In addition, HFBu diet‐fed GPR41^−/−^ and GPR43^−/−^ male mice were run in parallel with the WT mice on the HFBu diet. Body weight and food intake were measured on the last day of each week. After 15 weeks of intervention, the metabolic parameters were measured using metabolic cages. Mice were euthanized after 21 weeks of feeding. Blood serum, liver, epididymal fat, and subcutaneous fat were collected and stored at −80 °C for further analyses.

### Metabolic Cages

Mice were individually placed in metabolic cages (Comprehensive Laboratory Animal Monitoring System, CLAMS, USA) with free access to food and water. Room temperature for all metabolic studies was maintained at 25 °C to keep mice at thermoneutrality with a 12‐h light/dark cycle. After 12 h of acclimatization, VO2, VCO2, and energy expenditure were measured for 24 h spanning a single light–dark cycle. The oxygen consumption and carbon dioxide production rate normalized to animal body mass were displayed in the volume of gas consumption/production per kilogram of mass body weight per hour. Carbohydrate oxidation was calculated using the formula [(4.585 × VCO2) − (3.226 × VO2) ] × 4, in which the 4 represents the conversion from mass per time unit to kcal per time unit. Similarly, fat oxidation was calculated using the formula [(1.695 × VO2) − (1.701 × VCO2) ] × 9.^[^ ^68^ ^]^

### Insulin Sensitivity Analysis

According to the manufacturer's recommended protocol, plasma glucose was measured using an Accu‐Check Active Blood Glucose Meter (Accu‐Check Active, Roche Diagnostics). Insulin levels were measured using an enzyme‐linked immunosorbent assay using Ultra‐Sensitive Mouse Insulin ELISA Kit (90080, Crystal Chem Inc., USA). The degree of insulin resistance was estimated through a HOMA‐IR, which was calculated as the product of the fasting insulin level (mIU L^−1^) and the fasting glucose level (mmol L^−1^) divided by 22.5.^[^ ^69^ ^]^

### Biochemical Analysis

The serum AST, ALT, triglyceride, cholesterol, and low‐density‐lipoprotein levels were determined by an automatic biochemical analyzer (Cobas 701, Roche Diagnostics). All measurements were performed according to the manufacturer's instructions.

### Histological Analysis

Oil Red O staining was used to examine hepatic lipid accumulation as described previously.^[^ ^70^ ^]^ Briefly, frozen liver sections (10 µm) were stained with 0.5% Oil Red O (Sigma‐Aldrich) for 15 min and then washed with deionized Water. Next, paraffin‐embedded liver sections (5 µm) were stained with hematoxylin and eosin for the 30 s each to determine the liver damage. Three fields from three sections of each mouse were viewed under a Leica microscope and digitally photographed.

### RNA Extraction and Quantitative (q) Real‐Time PCR (RT‐PCR)

Total RNA was extracted from the liver with RNA Isolator Total RNA Extraction Reagent (Vazyme, China) before reverse‐transcription to cDNA using a high‐capacity cDNA reverse transcription kit (Takara, Japan). RT‐PCR was performed using the TransStart Top Green qPCR SuperMix (TransGen, China) and determined with a real‐time PCR detection system (Roche LightCycler480, Switzerland). The mRNA levels for specific genes were calculated using the formula 2 (−ΔΔCt) and normalized by β‐actin mRNA levels. All primers were listed in Table S2, Supporting Information.

### Western Blotting

Proteins were extracted from liver tissue or hepatocytes in cell lysis buffer (containing RIPA, Protease Inhibitor Cocktail, and 1 mM PMSF). After quantification using a BCA assay, 40–80 µg of protein were separated using 10% SDS–PAGE and then electro‐transferred to polyvinylidene difluoride (PVDF) membrane. Western blot assays were performed using primary antibodies. Antibodies against CPT1a (D3B3) (#12252S), Acc1 (#4190S), CaMKII (pan) (#3362), p‐CaMKII (Thr286) (#12716), CREB (48H2) (#91917), and p‐CREB (Ser133) (#9198) were provided by Cell Signaling Technology Co., Ltd. (USA). Antibodies against Srebp1 (WL02093), PPARα (WL00978), HDAC1 (WL01297), and HDAC2 (WL03149) were provided by Shenyang Wanlei Biological Technology Co., Ltd. (China). Antibody against β‐actin (AC026) was provided by ABclonal Biotechnology Co., Ltd. (China). Immunodetection was performed using Clarity ECL western blot substrate (Bio‐Rad, USA) and visualized with the ChemiDoc Touch imaging system (Bio‐Rad, USA). All quantitative analyses for total and cytosolic proteins were normalized to β‐actin.

### Statistical Analysis

The data were presented as the mean ± SEM. Data were analyzed using the statistical package SPSS (Version 20, Chicago, IL, USA). Statistical significance was established at *p* < 0.05. For studies including two groups, statistical differences between groups were calculated using a two‐tailed, unpaired Student's *t*‐test. For studies including three or four groups, differences between groups were determined using a one‐way analysis of variance test (ANOVA) followed by the post hoc Tukey test. Two‐way ANOVA was used to analyze the effects of multiple factors, such as treatment, genotype, and their interaction. If the main and interactive effects were defined, post‐hoc comparisons were performed using Tukey's multiple comparisons test to assess the statistical significance among groups.

## Conflict of Interest

The authors declare no conflict of interest.

## Author Contributions

M.Z. and X.Y. contributed equally to this work.

Conceptualization, X.Y. and Y.Y.; methodology, M.Z.; validation, M.Z., Y.G., Q.W., N.P., X.G., T.H., and H.H.; formal analysis, M.Z., X.G., T.H., and N.P.; investigation, X.Y.; data curation, M.Z.; writing—original draft preparation, M.Z. and X.Y.; writing—review and editing, Y.Y., X.‐F.H., and K.Z.; visualization, Y.Y.; and funding acquisition, X.Y. and Y.Y.

## Supporting information

Supplementary information

## Data Availability

All data needed to evaluate the conclusions in the paper are present in the paper and/or the Supplementary Materials. Additional data related to this paper may be requested from the authors.

## References

[1] J. G. Fan , S. U. Kim , V. W. Wong , J. Hepatol. 2017, 67, 862.28642059

[2] N. Chalasani , Z. Younossi , J. E. Lavine , A. M. Diehl , E. M. Brunt , K. Cusi , M. Charlton , A. J. Sanyal , Gastroenterology 2012, 142, 1592. 22656328 10.1053/j.gastro.2012.04.001

[3] S. Schuster , D. Cabrera , M. Arrese , A. E. Feldstein , Nat. Rev. Gastroenterol. Hepatol. 2018, 15, 349. 29740166 10.1038/s41575-018-0009-6

[4] M. P. Moore , R. P. Cunningham , R. J. Dashek , J. M. Mucinski , R. S. Rector , Obesity (Silver Spring) 2020, 28, 1843. 32893456 10.1002/oby.22964 PMC7511422

[5] S. E. Pryde , S. H. Duncan , G. L. Hold , C. S. Stewart , H. J. Flint , FEMS Microbiol. Lett. 2002, 217, 133. 12480096 10.1111/j.1574-6968.2002.tb11467.x

[6] G. den Besten , A. Bleeker , A. Gerding , K. van Eunen , R. Havinga , T. H. van Dijk , M. H. Oosterveer , J. W. Jonker , A. K. Groen , D. J. Reijngoud , B. M. Bakker , Diabetes 2015, 64, 2398. 25695945 10.2337/db14-1213

[7] Z. Li , C. Yi , S. Katiraei , S. Kooijman , E. Zhou , C. Chung , Y. Gao , J. van den Heuvel , O. Meijer , J. Berbée , M. Heijink , M. Giera , K. Willems van Dijk , A. Groen , P. Rensen , Y. Wang , Gut 2018, 67, 1269. 29101261 10.1136/gutjnl-2017-314050

[8] T. Yang , H. Yang , C. Heng , H. Wang , S. Chen , Y. Hu , Z. Jiang , Q. Yu , Z. Wang , S. Qian , J. Wang , T. Wang , L. Du , Q. Lu , X. Yin , Food Funct. 2020, 11, 10675. 33216087 10.1039/d0fo01954b

[9] D. Zhou , Y. W. Chen , Z. H. Zhao , R. X. Yang , F. Z. Xin , X. L. Liu , Q. Pan , H. Zhou , J. G. Fan , Exp. Mol. Med. 2018, 50, 1. 10.1038/s12276-018-0183-1 PMC6277380 30510243

[10] E. P. J. G. Neis , J. G. Bloemen , S. S. Rensen , J. R. van der Vorst , M. A. van den Broek , K. Venema , W. A. Buurman , C. H. C. Dejong , PLoS One 2016, 11,e0166161. 27835668 10.1371/journal.pone.0166161 PMC5105994

[11] J. G. Bloemen , K. Venema , M. C. van de Poll , S. W. Olde Damink , W. A. Buurman , C. H. Dejong , Clin. Nutr. 2009, 28, 657. 19523724 10.1016/j.clnu.2009.05.011

[12] A. J. Brown , S. M. Goldsworthy , A. A. Barnes , M. M. Eilert , L. Tcheang , D. Daniels , A. I. Muir , M. J. Wigglesworth , I. Kinghorn , N. J. Fraser , N. B. Pike , J. C. Strum , K. M. Steplewski , P. R. Murdock , J. C. Holder , F. H. Marshall , P. G. Szekeres , S. Wilson , D. M. Ignar , S. M. Foord , A. Wise , S. J. Dowell , J. Biol. Chem. 2003, 278, 11312. 12496283 10.1074/jbc.M211609200

[13] Y. H. Hong , Y. Nishimura , D. Hishikawa , H. Tsuzuki , H. Miyahara , C. Gotoh , K. C. Choi , D. D. Feng , C. Chen , H. G. Lee , K. Katoh , S. G. Roh , S. Sasaki , Endocrinology 2005, 146, 5092. 16123168 10.1210/en.2005-0545

[14] K. M. Maslowski , A. T. Vieira , A. Ng , J. Kranich , F. Sierro , D. Yu , H. C. Schilter , M. S. Rolph , F. Mackay , D. Artis , R. J. Xavier , M. M. Teixeira , C. R. Mackay , Nature 2009, 461, 1282. 19865172 10.1038/nature08530 PMC3256734

[15] Z. Ang , J. L. Ding , Front. Immunol. 2016, 7, 28. 26870043 10.3389/fimmu.2016.00028 PMC4734206

[16] Y. Xiong , N. Miyamoto , K. Shibata , M. A. Valasek , T. Motoike , R. M. Kedzierski , M. Yanagisawa , Proc. Natl. Acad. Sci. U S A 2004, 101, 1045. 14722361 10.1073/pnas.2637002100 PMC327148

[17] W. Q. Zhang , T. T. Zhao , D. K. Gui , C. L. Gao , J. L. Gu , W. J. Gan , W. Huang , Y. Xu , H. Zhou , W. N. Chen , Z. L. Liu , Y. H. Xu , J. Agric. Food Chem. 2019, 67, 7694. 31250637 10.1021/acs.jafc.9b02083

[18] A. Brown , S. Goldsworthy , A. Barnes , M. Eilert , L. Tcheang , D. Daniels , A. Muir , M. Wigglesworth , I. Kinghorn , N. Fraser , N. Pike , J. Strum , K. Steplewski , P. Murdock , J. Holder , F. Marshall , P. Szekeres , S. Wilson , D. Ignar , S. Foord , A. Wise , S. J. T. J.o.b.c. Dowell , J. Biol. Chem. 2003, 278, 11312. 12496283 10.1074/jbc.M211609200

[19] J. R. Davie , J. Nutr. 2003, 133, 2485S. 12840228 10.1093/jn/133.7.2485S

[20] B. M. Moran , P. R. Flatt , A. M. McKillop , Acta Diabetol. 2016, 53, 177. 26739335 10.1007/s00592-015-0826-9

[21] M. H. Wong , A. B. Samal , M. Lee , J. Vlach , N. Novikov , A. Niedziela‐Majka , J. Y. Feng , D. O. Koltun , K. M. Brendza , H. J. Kwon , B. E. Schultz , R. Sakowicz , J. S. Saad , G. A. Papalia , J. Mol. Biol. 2019, 431, 1440. 30753871 10.1016/j.jmb.2019.02.001

[22] W. C. Hsu , H. N. Le , Y. J. Lin , M. C. Chen , T. F. Wang , C. C. Li , W. W. Kuo , B. Mahalakshmi , C. H. Singh , M. C. Chen , C. Y. Huang , J. Cell. Biochem. 2021, 122, 612. 33459431 10.1002/jcb.29892

[23] J. Zhao , B. Wang , X. Wang , X. Shang , Mol. Cell. Biochem. 2018, 448, 71. 29427172 10.1007/s11010-018-3314-z

[24] J. Han , E. Li , L. Chen , Y. Zhang , F. Wei , J. Liu , H. Deng , Y. Wang , Nature 2015, 524, 243. 26147081 10.1038/nature14557

[25] S. Herzig , S. Hedrick , I. Morantte , S. Koo , F. Galimi , M. Montminy , Nature 2003, 426, 190. 14614508 10.1038/nature02110

[26] W. H. Oddy , C. E. Herbison , P. Jacoby , G. L. Ambrosini , T. A. O'Sullivan , O. T. Ayonrinde , J. K. Olynyk , L. J. Black , L. J. Beilin , T. A. Mori , B. P. Hands , L. A. Adams , Am. J. Gastroenterol. 2013, 108, 778. 23545714 10.1038/ajg.2013.95

[27] F. M. Trovato , G. F. Martines , D. Catalano , Nutrire 2018, 43, 11.

[28] L. Gan , W. Xiang , B. Xie , L. Yu , Front Med. 2015, 9, 275. 26290284 10.1007/s11684-015-0410-2

[29] K. Cusi , Gastroenterology 2012, 142, 711. 22326434 10.1053/j.gastro.2012.02.003

[30] A. Moshfegh , J. Goldman , L. Cleveland , What We Eat in America. NHANES 2001–2002, USDA, Agricultural Research Service, Beltsville (MD) 2005.

[31] A. M. Stephen , M. M. J. Champ , S. J. Cloran , M. Fleith , L. van Lieshout , H. Mejborn , V. J. Burley , Nutr. Res. Rev. 2017, 30, 149. 28676135 10.1017/S095442241700004X

[32] H. J. Wang , Z. H. Wang , J. G. Zhang , W. W. Du , C. Su , J. Zhang , F. Y. Zhai , B. Zhang , Eur. J. Clin. Nutr. 2014, 68, 619. 24619105 10.1038/ejcn.2014.24

[33] A. Reynolds , J. Mann , J. Cummings , N. Winter , E. Mete , L. Te Morenga , Lancet 2019, 393, 434. 30638909 10.1016/S0140-6736(18)31809-9

[34] G. Musso , R. Gambino , F. De Michieli , M. Cassader , M. Rizzetto , M. Durazzo , E. Fagà , B. Silli , G. Pagano , Hepatology 2003, 37, 909. 12668986 10.1053/jhep.2003.50132

[35] R. S. O'Connor , L. Guo , S. Ghassemi , N. W. Snyder , A. J. Worth , L. Weng , Y. Kam , B. Philipson , S. Trefely , S. Nunez‐Cruz , I. A. Blair , C. H. June , M. C. Milone , Sci. Rep. 2018, 8, 6289. 29674640 10.1038/s41598-018-24676-6 PMC5908836

[36] M. Kasubuchi , S. Hasegawa , T. Hiramatsu , A. Ichimura , I. Kimura , Nutrients 2015, 7, 2839. 25875123 10.3390/nu7042839 PMC4425176

[37] M. Waldecker , T. Kautenburger , H. Daumann , C. Busch , D. Schrenk , J. Nutr. Biochem. 2008, 19, 587. 18061431 10.1016/j.jnutbio.2007.08.002

[38] L. Sealy , R. Chalkley , Cell 1978, 14, 115. 667928 10.1016/0092-8674(78)90306-9

[39] G. Canettieri , I. Morantte , E. Guzmán , H. Asahara , S. Herzig , S. D. Anderson , J. R. Yates, 3rd , M. Montminy , Nat. Struct. Biol. 2003, 10, 175. 12567184 10.1038/nsb895

[40] K. T. Velázquez , R. T. Enos , J. E. Bader , A. T. Sougiannis , M. S. Carson , I. Chatzistamou , J. A. Carson , P. S. Nagarkatti , M. Nagarkatti , E. A. Murphy , World J. Hepatol. 2019, 11, 619. 31528245 10.4254/wjh.v11.i8.619 PMC6717713

[41] C.‐Y. Lian , Z.‐Z. Zhai , Z.‐F. Li , L. Wang , Chem.‐Biol. Interact. 2020, 330, 109199. 32805210 10.1016/j.cbi.2020.109199

[42] X. Liu , Y. Li , D. Tobias , D. Wang , J. Manson , W. Willett , F. Hu , J. Nutr. 2018, 148, 1821. 30247611 10.1093/jn/nxy183 PMC6209808

[43] M. Alves‐Bezerra , D. E. Cohen , Compr. Physiol. 2017, 8, 1. 29357123 10.1002/cphy.c170012 PMC6376873

[44] G. Li , H. Su , Z. Zhou , W. Yao , PLoS One 2014, 9, e97342. 24840136 10.1371/journal.pone.0097342 PMC4026140

[45] B. Onal , S. D. Unudurthi , T. J. Hund , Front. Pharmacol. 2014, 5, 9. 24550832 10.3389/fphar.2014.00009 PMC3912431

[46] J. S. Joseph , K. Anand , S. T. Malindisa , O. F. Fagbohun , Diabetes Metab. Syndr. 2021, 15, 589. 33714133 10.1016/j.dsx.2021.02.037

[47] J. S. Joseph , A. O. Ayeleso , E. Mukwevho , Trans. R. Soc. S. Afr. 2018, 73, 193.

[48] Z. Ang , D. Xiong , M. Wu , J. L. Ding , FASEB J. 2018, 32, 289. 28883043 10.1096/fj.201700252RR PMC5731126

[49] M. M. Mihaylova , R. J. Shaw , Trends Endocrinol. Metab. 2013, 24, 48. 23062770 10.1016/j.tem.2012.09.003 PMC3532556

[50] Y. R. Liu , J. Q. Wang , Z. G. Huang , R. N. Chen , X. Cao , D. C. Zhu , H. X. Yu , X. R. Wang , H. Y. Zhou , Q. Xia , J. Li , Int. J. Mol. Med. 2021, 48, 131. 34013366 10.3892/ijmm.2021.4964 PMC8136123

[51] Y. Guo , X. Zhang , Z. Zhao , H. Lu , B. Ke , X. Ye , B. Wu , J. Ye , Acta Pharm. Sin. B 2020, 10, 825. 32528830 10.1016/j.apsb.2020.02.005 PMC7276689

[52] M. Haberland , M. Carrer , M. Mokalled , R. Montgomery , E. Olson , J. Biol. Chem. 2010, 285, 14663. 20190228 10.1074/jbc.M109.081679 PMC2863240

[53] T. T. T. Nguyen , Y. Zhang , E. Shang , C. Shu , C. Torrini , J. Zhao , E. Bianchetti , A. Mela , N. Humala , A. Mahajan , A. O. Harmanci , Z. Lei , M. Maienschein‐Cline , C. M. Quinzii , M. A. Westhoff , G. Karpel‐Massler , J. N. Bruce , P. Canoll , M. D. Siegelin , J. Clin. Invest. 2020, 130, 3699. 32315286 10.1172/JCI129049 PMC7324177

[54] M. C. P. Cleophas , T. O. Crişan , H. Lemmers , H. Toenhake‐Dijkstra , G. Fossati , T. L. Jansen , C. A. Dinarello , M. G. Netea , L. A. B. Joosten , Ann. Rheum. Dis. 2016, 75, 593. 25589513 10.1136/annrheumdis-2014-206258

[55] A.‐G. Wang , S.‐B. Seo , H.‐B. Moon , H.‐J. Shin , D. H. Kim , J.‐M. Kim , T.‐H. Lee , H. J. Kwon , D.‐Y. Yu , D.‐S. Lee , Biochem. Biophys. Res. Commun. 2005, 330, 461. 15796905 10.1016/j.bbrc.2005.02.179

[56] A. Morán‐Costoya , A. M. Proenza , M. Gianotti , I. Lladó , A. Valle , Antioxid. Redox Signal 2021, 35, 753. 33736456 10.1089/ars.2021.0044

[57] N. Bazhan , T. Jakovleva , N. Feofanova , E. Denisova , A. Dubinina , N. Sitnikova , E. Makarova , Cells 2019, 8, 1529. 31783664 10.3390/cells8121529 PMC6953068

[58] D. Sorrentino , S. Zhou , E. Kokkotou , P. J. T. A Berk , Am. J. Physiol. 1992, 263, G380. 1415550 10.1152/ajpgi.1992.263.3.G380

[59] N. Ståhlberg , E. Rico‐Bautista , R. Fisher , X. Wu , L. Cheung , A. Flores‐Morales , G. Tybring , G. Norstedt , P. J. E. Tollet‐Egnell , Endocrinology 2004, 145, 1972. 14684613 10.1210/en.2003-0874

[60] Z. Wang , M. Xu , Z. Hu , M. Hultström , E. Lai , Eur. J. Gastroenterol. Hepatol. 2014, 26, 1015. 25003744 10.1097/MEG.0000000000000151

[61] M. T. Long , A. Pedley , J. M. Massaro , U. Hoffmann , J. Ma , R. Loomba , R. T. Chung , E. J. Benjamin , Liver Int. 2018, 38, 1495. 29377504 10.1111/liv.13709 PMC6206437

[62] E. Buzzetti , P. Parikh , A. Gerussi , E. J. P. Tsochatzis , Pharmacol. Res. 2017, 120, 97. 28336373 10.1016/j.phrs.2017.03.014

[63] A. Lonardo , F. Nascimbeni , S. Ballestri , D. Fairweather , S. Win , T. Than , M. Abdelmalek , A. J. H. Suzuki , Hepatology 2019, 70, 1457. 30924946 10.1002/hep.30626 PMC6766425

[64] S. Della Torre , V. Benedusi , R. Fontana , A. J. N.r.E. Maggi , Nat. Rev. Endocrinol. 2014, 10, 13. 24146033 10.1038/nrendo.2013.203

[65] S. J. F Della Torre , Front. Endocrinol. (Lausanne) 2020, 11, 572490. 33071979 10.3389/fendo.2020.572490 PMC7531579

[66] M. Matsumoto , W. Ogawa , K. Teshigawara , H. Inoue , K. Miyake , H. Sakaue , M. Kasuga , Diabetes 2002, 51, 1672. 12031952 10.2337/diabetes.51.6.1672

[67] H. Shi , Y. Yu , D. Lin , P. Zheng , P. Zhang , M. Hu , Q. Wang , W. Pan , X. Yang , T. Hu , Q. Li , R. Tang , F. Zhou , K. Zheng , X.‐F. Huang , Microbiome 2020, 8, 143. 33008466 10.1186/s40168-020-00920-y PMC7532656

[68] M. Schilperoort , A. D. van Dam , G. Hoeke , I. G. Shabalina , A. Okolo , A. C. Hanyaloglu , L. H. Dib , I. M. Mol , N. Caengprasath , Y. W. Chan , S. Damak , A. R. Miller , T. Coskun , B. Shimpukade , T. Ulven , S. Kooijman , P. C. Rensen , M. Christian , EMBO Mol. Med. 2018, 10, 3. 10.15252/emmm.201708047 PMC5840546 29343498

[69] D. R. Matthews , J. P. Hosker , A. S. Rudenski , B. A. Naylor , D. F. Treacher , R. C. Turner , Diabetologia 1985, 28, 412. 3899825 10.1007/BF00280883

[70] T. Kudo , T. Tamagawa , M. Kawashima , N. Mito , S. Shibata , J. Biol. Rhythms 2007, 22, 312. 17660448 10.1177/0748730407302625

