# Supplementary material for: Butyrate Attenuates Hepatic Steatosis Induced by a High‐Fat and Fiber‐Deficient Diet via the Hepatic GPR41/43‐CaMKII/HDAC1‐CREB Pathway
Source: Mol Nutr Food Res. 2022 Nov 16;67(1):2200597. doi: 10.1002/mnfr.202200597 (PMC10078002; doi:10.1002/mnfr.202200597)
Supplement: Supplementary file 1 — Supplementary information [file MNFR-67-0-s001.pdf]

Supporting Information

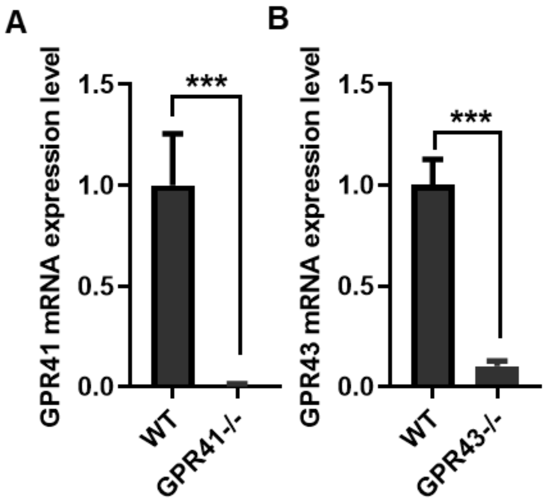

Figure S1. GPR41 and GPR43 receptors were knockout in the primary hepatocytes isolated from the liver tissue of GPR41$^{-/-}$ and GPR43$^{-/-}$ mice. A, B The mRNA expression of corresponding GPR41 and GPR43 receptors in the primary hepatocytes isolated from the liver tissue of GPR41$^{-/-}$ (A) and GPR43$^{-/-}$ (B) mice compared to the wildtype (WT) mice. Values are means ± SEM (n=6), ***$P < 0.001$

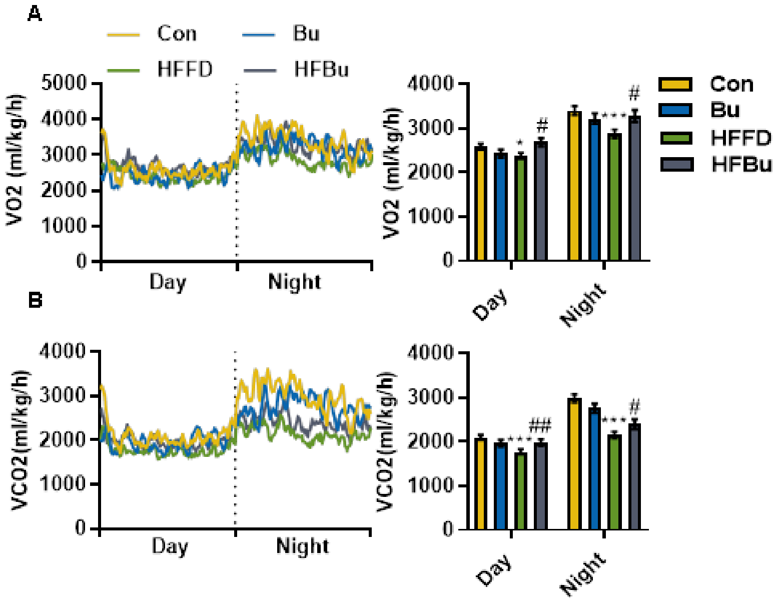

Figure S2. Sodium butyrate increased oxygen consumption and carbon dioxide production. A, B The mice were housed in fully automated metabolic cages in which Oxygen consumption (VO2) (A) and carbon dioxide production (VCO2) (B) were measured (n=10). Values are means ± SEM, **P* < 0.05, ****P* < 0.001 versus Con mice; $^{\#}P$< 0.05, $^{\#\#}P$ < 0.01 versus HFFD mice. Con, control diet (Lab chow diet); Bu, a diet of sodium butyrate mixed into lab chow diet; HFFD, high-fat fiber-deficient diet; HFBu, a diet of sodium butyrate mixed into HFFD diet.

Supplementary Table 1. Composition of the diets.

	Con	Bu		HFFD	HFBu
**Energy (%)**					
**Fat**	**13.1**	**13.1**		**55.2**	**55.2**
**Carbohydrate**	**58.2**	**58.2**		**30.1**	**30.1**
**Protein**	**28.7**	**28.7**		**14.7**	**14.7**
**Fiber (% by weight)**	**20**	**19**		**5**	**4.75**
**Sodium butyrate (% by weight)**	**0**	**5**		**0**	**5**
#Chemical composition (%)			Ingredient (% by weight)		
Nitrogen-free extract	50	47.5			
Starch	29.4	27.9	Corn Starch	19.3	18.2
Sucrose+glucose+fructose	1.16+0.23+0.27 (total 1.66)	1.10+0.22+0.26 (total 1.58)	Sucrose	19.2	18.2
Fat	5	4.8	Lard oil	31.5	29.9
Protein	24.6	23.4	Gelatine	5	4.8
			Methionine	0.3	0.3
			Caesin	13	12.4
Fibre	15	14.3	Cellulose	5.1	4.8
Minerals	6.1	6.1	Mineral Mix	5	4.7
Various vitamins	0.3-2200pm	0.3-2200pm	Vitamin Mix	1.3	1.2
Sodium butyrate	0	5	Sodium butyrate	0	5

Control (Con) diet was a lab chow diet (LabDiet 5010) with 20% dietary fiber by weight from plant polysaccharides, containing high microbiota-accessible carbohydrates derived from a diverse source of plants, including corn, soybean, wheat, oats, alfalfa, and beet; Bu was a diet mixed 50 g/kg sodium butyrate into lab chow diet (5% sodium butyrate by weight). HFFD was a high-fat (315 g/kg from fat, 31.5% by weight) and fiber-deficient (50g/kg from cellulose, 5% by weight). HFBu was a diet of sodium butyrate mixed into the HFFD diet (5% sodium butyrate by weight). Foods were made from semi-synthetic materials according to the recommendation of "AIN93 Diet for Laboratory Rodents".

#Chemical composition (%) of Con diet (LabDiet 5010) is calculated from the natural ingredients, including ground corn, dehulled soybean meal, wheat middlings, fish meal, whole wheat, wheat germ, brewers dried yeast, ground oats, dehydrated alfalfa meal, porcine animal fat, ground soybean hulls, calcium carbonate, dried beet pulp, salt, soybean oil, DL-methionine, pyridoxine hydrochloride, choline chloride, menadione dimethylpyrimidinol bisulfite (source of vitamin K), thiamine mononitrate, cholecalciferol, dicalcium phosphate, silicon dioxide, vitamin A acetate, folic acid, biotin, dl-alpha tocopheryl acetate, calcium pantothenate, riboflavin, nicotinic acid, vitamin B12, manganous oxide, zinc oxide, ferrous carbonate, copper sulfate, zinc sulfate, calcium iodate, cobalt carbonate.

Supplementary Table 2. Primers used for PCR.

Mouse Gene	Forward Primer (5'→3')	Reverse Primer (5'→3')
Acc1	TGCTGGATTATCTTGGCTTCA	CCCGTGGGAGTAGTTGCTGTA
Fasn	TCGGAGACAATTCACCAAACC	AGCCATCCCACAGGAGAAACC
Scd1	CTTCCTCCTGAATACATCCCTCC	CTCCATCCCATCTAGCACAACCT
Srebp1c	CTTGTTGCCTCCTCTTTTGCTTA	CTTTATTTCTCTCAATGACCCGTAG
PPARγ	CGATCTGCCTGAGGTCTG	GAGCCTAAGTTTGAGTTTGC
CPT1a	TATGGTCAAGGTCTTCTCGGGTCG	AGTGCTGTCATGCGTTGGAAGTCTC
Cyp4a10	GCAAACCATACCCAATCC	TCCCAAGTGCCTTTCCTA
Cyp4a14	AGCAATTCAAAGCGGAGC	GCCTACATCATAACCCACG
Mcad	TAACATACTCGTCACCCTTC	ATGCCTGTGATTCTTGCT
GPR41	ACTGGCTTTTCTTTTCCGTCTA	CATGCGAAACGGCAGGAAGA
GPR43	GCGGACTTGCTCCTGTTGC	CAGCCACGTGCTGCAGTAGA
β-actin	TGAGAGGGAAATCGTGCGTGAC	GCTCGTTGCCAATAGTGATGACC
